# Impact of Phosphate Availability on Membrane Lipid Content of the Model Strains, *Streptomyces lividans* and *Streptomyces coelicolor*

**DOI:** 10.3389/fmicb.2021.623919

**Published:** 2021-02-22

**Authors:** Clara Lejeune, Sonia Abreu, Pierre Chaminade, Thierry Dulermo, Michelle David, Sebastiaan Werten, Marie-Joelle Virolle

**Affiliations:** ^1^CEA, CNRS, Institute for Integrative Biology of the Cell (I2BC), Université Paris-Saclay, Gif-sur-Yvette, France; ^2^Lipides, Systèmes Analytiques et Biologiques, Université Paris-Saclay, Châtenay-Malabry, France; ^3^Lesaffre International, Marcq-en-Baroeul, France; ^4^Institute of Biological Chemistry, Biocenter, Medical University of Innsbruck, Innsbruck, Austria

**Keywords:** phospholipids, ornithine lipids, triacylglycerol, acyltransferase, hemolysin, phospholipase C, phosphatidylinositol, phosphate limitation

## Abstract

In this issue we demonstrated that the phospholipid content of *Streptomyces lividans* varies greatly with Pi availability being was much lower in Pi limitation than in Pi proficiency whereas that of *Streptomyces coelicolor* varied little with Pi availability. In contrast the content in phosphate free ornithine lipids was enhanced in both strains in condition of phosphate limitation. Ornithine lipids biosynthesis starts with the *N*-acylation of ornithine to form lyso-ornithine that is then *O*-acylated to yield ornithine lipid. The operon *sco1222-23* was proposed to be involved in the conversion of specific amino acids into ornithine in condition of phosphate limitation whereas the *sco0921-20* operon encoding *N*- and *O*-acyltransferase, respectively, was shown to be involved in the biosynthesis of these lipids. The expression of these two operons was shown to be under the positive control of the two components system PhoR/PhoP and thus induced in phosphate limitation. The expression of *phoR/phoP* being weak in *S. coelicolor*, the poor expression of these operons resulted into a fivefold lower ornithine lipids content in this strain compared to *S. lividans*. In the deletion mutant of the *sco0921-20* operon of *S. lividans*, lyso-ornithine and ornithine lipids were barely detectable and TAG content was enhanced. The complementation of this mutant by the *sco0921-20* operon or by *sco0920* alone restored ornithine lipids and TAG content to wild type level and was correlated with a twofold increase in the cardiolipin content. This suggested that SCO0920 bears, besides its broad *O*-acyltransferase activity, an *N*-acyltransferase activity and this was confirmed by the detection of lyso-ornithine in this strain. In contrast, the complementation of the mutant by *sco0921* alone had no impact on ornithine lipids, TAG nor cardiolipin content but was correlated with a high lyso-ornithine content. This confirmed that SCO0921 is a strict *N*-acyltransferase. However, interestingly, the over-expression of the *sco0921-20* operon or of *sco0921* alone in *S. coelicolor*, led to an almost total disappearance of phosphatidylinositol that was correlated with an enhanced DAG and TAG content. This suggested that SCO0921 also acts as a phospholipase C, degrading phosphatidylinositol to indirectly supply of phosphate in condition of phosphate limitation.

## Introduction

In *Streptomyces* species, as in other bacteria, the phospholipids of the bacterial membrane constitute a major phosphorus reservoir and, in condition of phosphate deprivation, bacteria have evolved various strategies to replace their phospholipids by phosphorus-free lipids in order to retrieve and save phosphate. Phosphorus-free lipids include glycolipids, amino lipids (ornithine, glycine, lysine, glutamine, serine…), sulfolipids, and betaine lipids (Benning et al., 1995; Geiger et al., 1999; Lewenza et al., 2011; Diercks et al., 2015; Sohlenkamp, 2016; Lev et al., 2019). In *Streptomyces*, the most common of these lipids are ornithine lipids (OL) whose synthesis requires two enzymatic steps (Sandoval-Calderón et al., 2015, 2017). The first step was proposed to be catalyzed by SCO0921 (OlsB), a supposedly very specific *N*-acyltransferase that transfers the first acyl chain onto the NH_2_ group of ornithine to yield lyso-ornithine (Weissenmayer et al., 2002; Gao et al., 2004; Sandoval-Calderón et al., 2015; Sohlenkamp, 2016). Subsequently, the *O*-acyltransferase SCO0920 (OlsA) was proposed to transfer the second acyl chain onto the hydroxyl group of the acyl moiety of the lyso-ornithine lipid (LOL) to yield OL (Weissenmayer et al., 2002; Gao et al., 2004; Sandoval-Calderón et al., 2015; Sohlenkamp, 2016). In many *Streptomyces* species including *Streptomyces coelicolor* (*SC*) and *Streptomyces lividans* (*SL*), these two genes form an operon under the positive control of the two component system (TCS) PhoR/PhoP (Weissenmayer et al., 2002; Gao et al., 2004; Martín et al., 2012; Sandoval-Calderón et al., 2015).

*Streptomyces coelicolor* and *S. lividans* are phylogenetically very closely related model strains showing nonetheless very contrasted metabolic features (Esnault et al., 2017). *S. lividans* is a weak antibiotic producer characterized by a glycolytic metabolism promoting triacylglycerol (TAG) accumulation whereas *S. coelicolor* is a strong antibiotic producer characterized by an oxidative metabolism incompatible with TAG accumulation (Esnault et al., 2017). Recent studies indicate that these drastically different metabolic features are, at least in part, due to the weaker expression of the two components system PhoR/PhoP in *S. coelicolor* compared to *S. lividans* (Millan-Oropeza et al., 2020). This TCS governs the adaptation of the bacteria to low phosphate availability *via* the positive and negative control it exerts on phosphate and nitrogen assimilation, respectively (Martín et al., 2017). The consequences of low and high phosphate (Pi) availability on the lipid composition of the membranes of these two model strains was assessed and revealed that the phospholipids content was reduced in condition of Pi limitation in *S. lividans* whereas that of *S. coelicolor* varies little with phosphate availability. In contrast the OL content was enhanced in condition of Pi limitation in both strains. Ornithine biosynthesis was previously shown to involve the *sco0921-20* operon whose expression is under the positive control of PhoR/PhoP and thus induced in condition of phosphate limitation (Martín et al., 2012; Sandoval-Calderón et al., 2015) and our data confirmed this statement. Furthermore, we identified an operon (*sco1222-23*) encoding enzymes putatively involved in the conversion of arginine, proline and glutamate into ornithine in condition of phosphate limitation and we demonstrated that the expression of this operon was also under the positive control of PhoR/PhoP. At last, we assessed the consequences of deletion or over-expression of the *sco0921-20* operon on the lipidome of *S. lividans* and *S. coelicolor*. The complementation of the *sco0921-20* operon deletion mutants by an ectopic copy of this operon, or by *sco0920* or *sco0921* alone, gave clue of unexpected additional functions of these two enzymes.

## Materials and Methods

### Bacterial Strains, Media and Culture Conditions

Bacteria strains used in this study were *S. lividans* TK24 (*SL*, Ruckert, 2015) and *S. coelicolor* M145 (*SC*, Bentley, 2002). These strains were grown on SFM medium (Kieser et al., 2000) to obtain spores. Modified R2YE agar medium (Kieser et al., 2000), devoided of sucrose and with no phosphate added (condition of phosphate limitation, 1 mM free phosphate final as determined by PiBlueTM test from BioAssay Systems) and supplemented with glucose 50 mM, was used for solid-grown cultures of these strains. 10^6^ spores of each strain were plated on the surface of cellophane disks (Focus Packaging & Design Ltd, Louth, United Kingdom) laid down on the surface of R2YE agar medium and incubated at 28°C in darkness for 72 h. Mycelial lawns of each of the four replicate of the different strains were collected with a spatula, washed twice with deionized water, lyophilized and weighted.

### Lipid Extraction and Characterization by LC/Corona-CAD and LC/MS

Lipid extraction was performed by a procedure derived from Folch’s method (Folch et al., 1957) from four independent cultures of *Streptomyces antibioticus* DSM 41,481 and DSM 40,868. A defined volume (4.5 mL) of chloroform/methanol (1:2) was added to 10 mg of lyophilized *Streptomyces* mycelium and vortexed for 30 s. The mixture was left at ambient temperature for 1 h, then 1.25 mL of water was added, and the mixture was vortexed for 30 s. The mixture was then centrifuged (1000 × *g* for 10 min) to obtain phase separation. The lower organic phase was collected, and the upper aqueous phase was submitted to a second extraction by adding 2 mL of chloroform/methanol (85:15). The two organic phases were pooled and evaporated under a stream of nitrogen at room temperature. The dry residue was dissolved in 400 μL of isooctane/chloroform (4:1) before analysis. The chromatographic conditions have been described previously (Abreu et al., 2017). Briefly, lipid class analysis was performed with an Inertsil Silica (150 mm × 2.1 mm I.D, 5 μm) column (GL Sciences Inc., Tokyo, Japan) thermostated at 40°C. The HPLC instrumentation consisted of the system Dionex U-3000 RSLC (Thermofisher, Villebon, France). A quaternary solvent gradient was used to elute all the lipid classes present in the sample by increasing the order of polarity. Lipid class identification was verified by coupling the chromatographic separation to mass spectrometry. MS analyses were performed with a LTQ-Orbitrap Velos Pro (Thermo Fisher Scientific) equipped with an APPI ion source. The MS2 and MS3 spectra were obtained in data-dependent acquisition (DDA) mode. Lipid detection was performed using a Corona-CAD system (ESA, Chelmsford, MA, United States) (Abreu et al., 2017); the signal was acquired with a Chromeleon data station (Thermo Fisher Scientific, Villebon-sur-Yvette, France). Corona-CAD is a universal detector used for liquid chromatography and described in Dixon and Peterson (2002). The differences in the composition of the lipid classes in the samples are expressed as peak areas. All the data were subjected to Anova test using R 3.3.2 (R Core Team, 2013) and the “multcompView” package (Graves et al., 2019). The results obtained are presented as the mean ± standard error; a *p*-value <0.05 was considered as statistically significant. The letters above the histograms indicate the significance of the differences. When two histograms bear two different letters that mean that they are statistically significantly different but when they show the same letter that means that they are not statistically significantly different (*p* > 0,05; Tukey-adjusted comparisons).

Due to their low amount in samples, LOL could not be detected by LC-Corona-CAD analysis. They had to be detected and quantified using LC/MS by extracting their specific mass from the total ion current. The peak areas of [M-H]^–^ @*m/z* 371.3; 385.3; 399.3; 425.3 corresponding to C15:0, C16:0, C17:0, and C19:1 3-hydroxy fatty acids where summed up and shown in Figure 4.

### RNA Preparation and qRT-PCR Experiments

RNA was isolated from mycelia obtained from *S. lividans* TK24 (*SL*), wild type, its *phoP* mutant and *S. coelicolor* M145 (*SC*) grown for 40 h at 28°C on the solid R2YE medium with no K_2_HPO_4_ added (containing 1 mM free phosphate from elements of the media as determined with PiBlue^TM^ Phosphate Assay Kit from BioAssays Systems). In order to preserve RNA integrity, the mycelium was immediately freezed in liquid nitrogen in a solution containing denaturating guanidinium thiocyanate buffer RA1 (Macherey-Nagel, Hoerdt, France), phenol-chloroform and ß-mercaptoethanol (a reducing agent). The cells were then lysed and homogenized in the presence of glass beads (diameter < 106 μm) using a Fast-Prep apparatus (Savant Instruments). Total RNA was purified using the Nucleospin RNA Kit (Macherey-Nagel, Hoerdt, France), according to the manufacturer’s instructions. To remove residual DNA, a DNAse TURBO^TM^ treatment (Invitrogen) was performed at 37°C for one hour and total RNA was purified with the Nucleospin RNA Clean-Up kit (Macherey-Nagel, Hoerdt, France). The RNA concentrations were quantified using the Nanodrop 2000 spectrophotometer (Thermo Scientific). The integrity of the RNAs was verified using the Agilent 2100 bioanalyzer with the eukaryote total RNA 6000 Nano assay (Agilent Technologies). A total of 1 μg of total RNA was reverse transcribed in a 20 μL final reaction volume using the High Capacity cDNA Reverse Transcription Kit (Life Technologies) with RNase inhibitor and random primers following the manufacturer’s instructions. Quantitative PCR was performed on a QuantStudio 12K Flex Real-Time PCR System (Life Technologies) with a SYBR green detection protocol. A total of 3 ng of cDNA were mixed with Fast SYBR Green Master Mix and 750 nM of each primer in a final volume of 10 μL. The reaction mixture was loaded on 384 well microplates and submitted to 40 cycles of PCR (95°C/20 sec; [95°C/1 s; 60°C/20 s] X40) followed by a fusion cycle to analyze the melting curve of the PCR products. A qPCR analysis in the absence of a reverse transcription step was performed on all RNA samples to check the absence of any DNA contamination. Primers were designed using the Primer-Blast tool from NCBI and the Primer Express 3.0 software (Life Technologies) (Supplementary Table S1). Specificity and the absence of multi-locus matching at the primer site were verified by BLAST analysis. The amplification efficiencies of primers were generated using the slopes of standard curves obtained by a 10-fold dilution series. Amplification specificity for each real-time PCR reaction was confirmed by analysis of the dissociation curves. Each sample measurement was made in duplicate and four independent RNA biological samples were prepared for each condition. Determined cycle threshold (Ct) values were then exploited for further analysis. Cycle threshold is defined as the calculated cycle number at which the PCR product crosses the threshold of detection. This value tells how many cycles it took to detect the signal from your samples. Seven most stable reference genes were selected by GenEx software (MultiD) and the geometric mean of the five most stable genes (Glk/SCO2126, AspS/SCO3795, GyrA/SCO3873, GyrB/SCO3874, and RpoB/SCO4654) was used to normalize the data (HrdB/SCO5820 and RecG/SCO5566 were excluded). The determination of the relative gene expression ratio was achieved using the ΔΔCt method using three biological replicates (Pfaffl, 2001). The values of ΔΔCt of *SC* and of the *phoP* mutant of *SL* were normalized and standardized by log transforming, mean centering and autoscaling (Willems et al., 2008). All data were subjected to the Student test and the results were presented as the mean of delta-delta-Ct with error bars representing 95% confidence interval.

### Deletion and Over-Expression of the *sco0921-20* Operon or of *sco0920* or *sco0921* Alone in Wild Type and *sco0921-20* Deletion Mutants of *S. lividans* TK24 and *S. coelicolor* M145

All strains and plasmids used for these constructs are listed in Supplementary Table S2. DNA fragments were amplified by PCR from genomic DNA of *SL* and *SC* prepared as described previously (Sambrook and Russell, 2001), using primers listed in Supplementary Table S3. Primers used for PCR amplification and gene sequence analysis (Sci-Ed Software) were designed with Clone Manager Professional 9 software and purchased from IDT (Integrated DNA Technologies, Leuven, France). PCR amplifications were performed in a Techne ^3^Prime thermal cycler with Phusion High-Fidelity DNA polymerase (ThermoFisher Scientific, Illkirch Graffenstaden, France). PCR conditions were: 30 s at 98°C, then 10 s at 98°C for 30 cycles, followed by 30 s at 65°C then 6 min at 72°C. PCR fragments were purified with a NucleoSpin^®^ PCR clean-up Gel extraction Kit (Macherey-Nagel, Hoerdt, France).

In order to delete the *sco0921-20* operon in *SL* and *SC*, approximately 1 kb DNA fragments flanking this operon were amplified by PCR from genomic DNA of *SL* and *SC* prepared as described previously (Sambrook and Russell, 2001). The resulting PCR fragments were digested with adequate restriction enzymes, *HindIII* and *XbaI* for upstream region, *EcoRI* and *BamHI* for downstream region, and the apramycin resistance cassette (apra^*R*^, recovered from pW60 plasmid; Li et al., 1997) with *XbaI* and *EcoRI* restriction enzymes (ThermoFisher Scientific, Illkirch Graffenstaden, France). These three fragments were ligated into the plasmid pOSV400 (Boubakri et al., 2015) cut by the *HindIII* and *BamHI* sites, in presence of T4 DNA Ligase (5 U/μL, ThermoFisher Scientific, Illkirch Graffenstaden, France). The resulting plasmid was called pOSV400-UD-*sco0921-20-apra^*R*^*.

In order to over-express the *sco0921-20* operon or *sco0920* or *sco0921* alone, the amplified PCR fragments were purified and digested with *HindIII* and *PstI* (ThermoFisher Scientific, Illkirch Graffenstaden, France) were ligated behind the constitutive promoter *ermE*^∗^ (Siegl et al., 2013) into the plasmid pOSV557, a derivative of pOSV010 carrying the *ermE*^∗^ promoter (Juguet et al., 2009). The resulting three plasmids were called pOSV557-*sco0921-20*, pOSV557*-sco0921*, and pOSV557*-sco0920*.

The latter as well as pOSV400-UD-*sco0921-20-apra^*R*^* used for the deletion of the *sco0921-20* operon were transferred into competent *Escherichia coli* DH5α (Hanahan, 1983) by transformation using standard procedures (Kieser et al., 2000), purified and sequenced for structural verification. The plasmids were then transformed by electroporation into *E. coli* ET12567 pUZ8002 (Gust et al., 2004). The resulting *E. coli* ET12567 pUZ8002 strains were used to introduce the recombinant plasmids by conjugation into *SC* and *SL* using standard procedures (Kieser et al., 2000).

After 1 week of sporulation at 28°C, the ex-conjugants *sco0921-20* disruptive mutants, apramycin-resistant and hygromycin-sensitive, resulting from the transformation of pOSV400-UD-*sco0921-20-apra^*R*^*, were spotted on the solid medium HT (Kieser et al., 2000) containing apramycin (30 μg mL^–1^) and hygromycin (50 μg mL^–1^). The replacement of the *sco0921-20* operon by apra^*R*^ cassette in the genome of the mutant strains was confirmed by PCR using primers shown in Supplementary Table S3.

Concerning the three constructions used for over-expression, the hygromycin-resistant ex-conjugants were selected on the solid medium HT (Kieser et al., 2000) containing hygromycin (50 μg mL^–1^). The integration of the *sco0921-20* operon, of *sco0920* or of *sco0921* alone into the genome of the target strains was verified by PCR *via* amplification of the sequences surrounding the *attL* and *attR* sites using primers shown in Supplementary Table S3.

### Assay of Extracellular Actinorhodin (ACT) Production

Extracellular actinorhodin (ACT) was quantified from four individual plates of each strain grown on the conditions described above. After 72 h of incubation, mycelia were scrapped off the cellophane disks of each plate with a spatula and lyophilized in order to assess their mass. The R2YE agar medium (Kieser et al., 2000) present below the surface of cellophane disks was cut into small pieces and allowed to diffuse in 10 mL water for 2 h at 4°C. The first eluate was transferred into a new tube, and 10 mL of water was added again to the agar medium and allowed to diffuse for 2 h at 4°C. The second eluate was pooled with the first eluate, and 10 mL of water was added again to the agar medium and allowed to diffuse for 1 h at 4°C. The last eluates were pooled to the other two (30 mL final). Three mL of HCl (3 M) were added to 6 mL of the final eluate. The mixture was incubated on ice for one night to allow ACT precipitation. Precipitated ACT was collected by centrifugation (13,000 *g* for 30 min). Supernatants were discarded and the ACT pellets were suspended in 1 mL of KOH 1 M. Optical density of the solution was determined at 640 nm in a Shimadzu UV-1800 spectrophotometer using KOH 1 M as blank (Kieser et al., 2000).

### Protein Sequence Analysis

The Conserved Domain Database (Lu et al., 2020) was used to detect known functional domains. Alignment of SCO0921 with representative members of COG3176, a family of putative hemolysins, was performed with MAFFT (Katoh et al., 2019).

## Results

### Impact of Phosphate Availability on the Lipidome of *S. lividans* and *S. coelicolor*

**FIGURE 1:**
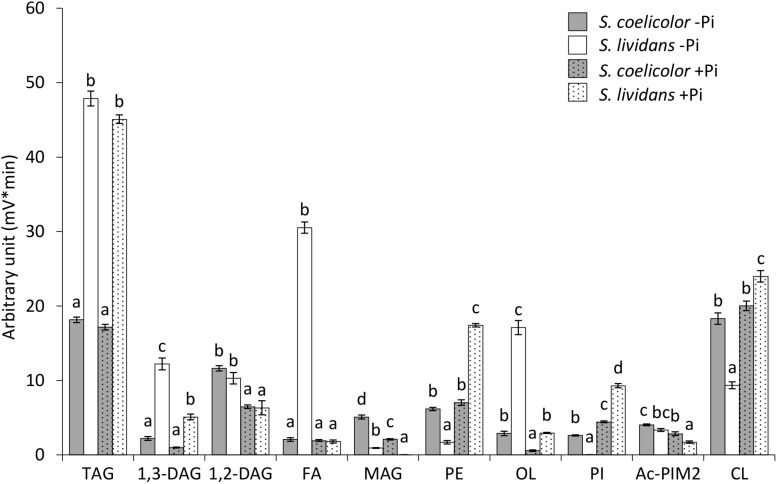
LC/Corona-CAD analysis of the total lipid content of *S. lividans* TK24 (white histograms) and *S. coelicolor* M145 (gray histograms) grown for 72 h at 28°C on solid R2YE medium with glucose (50 mM) as the main carbon source and containing either 1 mM (Pi limitation, plain histograms) or 5 mM phosphate (Pi proficiency, dotted histograms).TAG, triacylglycerol; DAG, diacylglycerol (1,2 or 1,3); FA, fatty acids; MAG, monoacylglycerol; PE, phosphatidylethanolamine; OL, ornithine lipids; PI, phosphatidylinositol; Ac-PIM2, acetylated phosphatidylinositol mannoside 2; CL, cardiolipid. Means values are shown as histograms with error bars representing standard error. Means sharing a letter are not significantly different (*P* > 0.05; Tukey-adjusted comparisons).

In order to assess the impact of phosphate availability on the lipid and fatty acid content of *SL* and *SC*, cultures of these strains grown for 72 h on solid R2YE limited or proficient in phosphate was analyzed by LC/Corona-CAD (Abreu et al., 2017). The results are shown in Figure 1.

In *S. lividans* (*SL*), the content in the phospholipids, phosphatidyl inositol (PI), phosphatidyl ethanolamine (PE) and cardiolipin (CL) was, respectively, 189, 8.8, and 2.4 fold higher in Pi proficiency than in Pi limitation. In contrast in *S. coelicolor* (*SC*) the content in PE and CL did not change with Pi availability whereas the PI content of *SC* was only 1.6 fold higher in Pi proficiency than in Pi limitation (versus 189 fold for *SL*). In Pi proficiency, PE, PI and CL were, respectively, 2.3, 2, and 1.1 fold more abundant in *SL* than in *SC* (Figures 1c and d). In Pi limitation, PI, PE and CL were, respectively, 53.5, 3.1, and 1.8 fold more abundant in *SC* than in *SL*. These data thus revealed a default of PL biosynthesis in *SC* even in Pi proficiency. This might be due to reduced Pi availability in *SC* linked to the weak expression of the genes of the Pho regulon involved in Pi supply in this strain (Millan-Oropeza et al., 2020). Furthermore, the 189 fold lower PI content of *SL* in condition of Pi limitation compared to Pi proficiency suggested that this very low PI content did not rely exclusively on reduced synthesis but also onto enhanced degradation of this specific phospholipid. The 53.5 higher PI content of *SC* compared to *SL* in Pi limitation suggested that the putative degradation of this specific phospholipid might be dependent of PhoR/PhoP that is known to be weakly expressed in *SC* (Millan-Oropeza et al., 2020). We will see later in the manuscript that SCO0921 might be involved in this process.

The higher PL content of *SL* in condition of Pi proficiency was accompanied by lower content of all biosynthetic intermediates FA (14.8 fold), MAG (22.1 fold), 1,2-DAG (1.7 fold), and 1,3-DAG (1.8 fold) in Pi proficiency than in Pi limitation. In *SC*, the content in MAG and DAG was also lower in Pi proficiency than in Pi limitation whereas that of the FA was similar in both Pi conditions. In Pi limitation, the FA content of *SL* was much higher (12.9 fold) than that of *SC*. This is consistent with the glycolytic (acetylCoA generating) versus oxidative (acetylCoA consuming) metabolism of *SL* and *SC*, respectively (Esnault et al., 2017). Furthermore, the abundance of free FA in *SL* in condition of Pi limitation, compared to the condition of Pi proficiency, might indicate insufficient glycerol 3P generation in Pi limitation as suggested by Millan-Oropeza et al. (2020) as well as too low Pi availability for PL biosynthesis. The MAG content was 4.8 and 50.3 fold higher in *SC* than in *SL* in Pi limitation and proficiency, respectively, whereas in contrast the 1,3-DAG content of *SC* was 4.6 and 4.4 fold lower than that of *SL* in Pi limitation and proficiency, respectively. This higher abundance of MAG in *SC* compared to *SL* in Pi proficiency, that is correlated with a lower abundance of 1,3 DAG, might be related to the inability of *SC* to synthetize PL in this condition. All biosynthetic intermediates MAG, 1,2-DAG and 1,3-DAG, were less abundant in Pi proficiency than in Pi limitation in both strains but these differences were stronger in *SL* than in *SC*. These differences might also be due to more active PL synthesis in Pi proficiency than in Pi limitation in *SL* compared to *SC*.

The OL content of *SL* was 5.1 and 4.3 fold higher than that of *SC* in Pi limitation and proficiency, respectively. This is consistent with the PhoR/PhoP dependent induction of OL biosynthesis (Sandoval-Calderón et al., 2015). Since PhoR/PhoP is known to be weakly expressed in *SC* (Millan-Oropeza et al., 2020), induction of OL synthesis is not as strong in *SC* as in *SL* and thus results in a lower OL content in *SC* than in *SL*. However, OL synthesis remains inducible in condition of Pi limitation in *SC* since OL content was 3.9 fold higher in Pi limitation than in Pi proficiency in *SC* (versus 5.3 in *SL*).

The TAG content was 2.5 fold higher in *SL* than in *SC* in both Pi conditions confirming the previously reported higher ability of *SL* to store TAG compared to *SC* (Esnault et al., 2017). This is consistent with the glycolytic (acetylCoA generating) versus oxidative (acetylCoA consuming) metabolism of *SL* and *SC*, respectively (Esnault et al., 2017). However, unexpectedly the TAG content of the strains did not vary with Pi availability.

Altogether our data indicated that *SC* had a lower phospholipid and TAG content than *SL* and that its lipid content varies little with Pi availability, in contrast to *SL*. This is thought to be due to the previously reported oxidative metabolism of this strain that consumes acetylCoA (Esnault et al., 2017) as well as to its severe phosphate limitation linked to the weak expression of the TCS PhoR/PhoP in this strain (Millan-Oropeza et al., 2020). Interestingly this study revealed that the phosphatidylinositol (PI) is the phospholipid bearing the greatest variation of content between the two strains and between the two Pi conditions. This suggested that this specific phospholipid might constitute a phosphate reserve.

### The *sco921-20* and *sco1222-23* Operons Are Involved in Ornithine Lipids Biosynthesis and Their Expression Is Under the Positive Control of PhoR/PhoP

**FIGURE 2:**
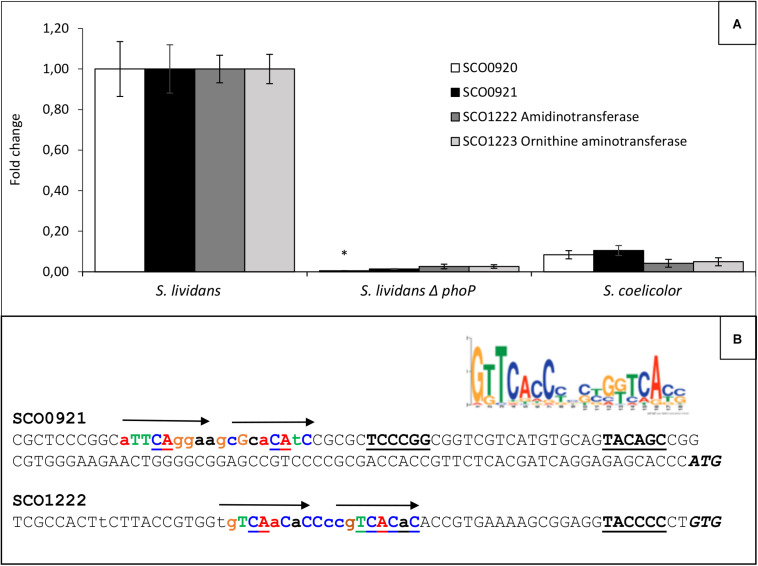
Determination in qRT-PCR of relative expression of genes of the *sco0921-0920* and *sco1222-1223* operons in *S. lividans* TK24, its *phoP* mutant and *S. coelicolor* M145 grown for 40 h at 28°C on solid R2YE, limited in phosphate (1 mM) and with glucose (50 mM) as main carbon source. **(A)** Level of expression of *sco0921* and *sco0920* (black and white histograms, respectively) and *sco1222* and *sco1223* (dark and light gray histograms, respectively) in the three strains. *S. lividans* TK24 was taken as reference equal to 1. Means values are shown as histograms with error bars representing 95% confidence interval. An unique asterisk signal significant abundance change. **(B)** Consensus PhoP box proposed by Allenby et al. (2012). Promoter regions of *sco0921* and *sco1222*. Putative – 10 and –35 promoter regions are in bold and underlined. Putative *pho* boxes are in bold colored letters and are represented by arrows above the sequence line. Upper and lower case letters represent most and least conserved bases compared to the consensus. The ATG and GTG translational start codons are in italic and bold letters.

Ornithine lipids are phosphate free lipids synthesized in condition of phosphate limitation to surrogate a fraction of phospholipids, in order to save phosphate (Martín et al., 2012; Sandoval-Calderón et al., 2015). In *Streptomyces*, their biosynthesis is known to rely on the expression of the *sco0921-20* operon (Sandoval-Calderón et al., 2015) proposed to encode *N*- and *O*-acyltransferase, respectively. The expression of this operon was shown to be under the positive control the TCS PhoR/PhoP in *S. coelicolor* (Martín et al., 2012; Sandoval-Calderón et al., 2015) as was the expression of related operons in other bacteria (Benning et al., 1995; Geiger et al., 1999; Barbosa et al., 2018; Lev et al., 2019) and our data confirmed this statement. However, since OL biosynthesis also requires ornithine availability, we search and identified in our *S. lividans* and *S. coelicolor* proteomic data, an operon, *sco1222-23*, encoding proteins, putatively involved in the conversion of specific amino acids (Arg, Pro, and Glu) into ornithine, whose expression was much lower in *S. coelicolor* than in *S. lividans* (Millan-Oropeza et al., 2020). This suggested that the expression of this operon was under the positive control of PhoR/PhoP and we confirmed that it was indeed the case (Figure 2A). *sco1223* encodes a ornithine aminotransferase, related to RocD of *Bacillis subtilis*, involved in the conversion of pyrroline-5-carboxylate, originating from glutamate or proline degradation, into ornithine. *sco1222* encodes a protein of the aminohydrolase family that is possibly involved into the degradation of arginine into ornithine. The expression of the first gene of the operon, *sco1222*, was reported to be stimulated by arginine, independently on the presence of the ArgR regulator (Pérez-Redondo et al., 2012). The induction of the expression of this operon by arginine might thus be mediated by SCO1221, a regulator of the AsnC-familly encoded by a gene located just upstream this operon. Putative Pho boxes were detected in the promoter region of the first genes of these two operons and are shown in Figure 2B. The expression of PhoR/PhoP being weak in *SC* compared to *SL* (Millan-Oropeza et al., 2020), the expression of these two operons is also weak in *SC* explaining the 5.1 fold lower OL content of this strain compared to *SL*, in phosphate limitation (Figure 1c).

### Impact of the Deletion of the *sco0921-20* Operon and Its Complementation by the *sco921-20* Operon, *sco0921* or *sco0920* Alone on the Lipidome of *S. lividans* and *S. coelicolor*

The *sco0921-20* operon was deleted in *SL* and *SC* and the deletion mutants were complemented by a unique ectopic copy of the *sco0921-20* operon, or by *sco0920* or *sco0921* alone. The expression of these genes was put under the control of the strong *ermE*^∗^ promoter (Siegl et al., 2013) and inserted at a unique position in the genome of the deletion mutant at the pSAM2 attB integration site (Boccard et al., 1989).

**FIGURE 3:**
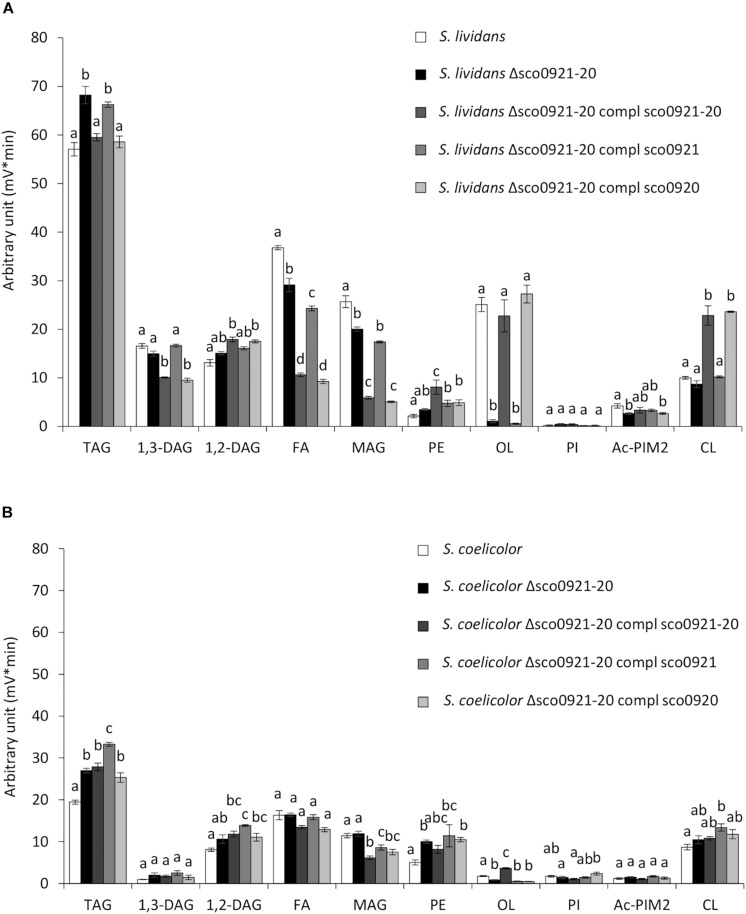
LC/Corona-CAD analysis of the total lipid content of the *wt* strains of *S. lividans* TK24 and *S. coelicolor* M145 and of derivatives of these strains deleted for the *sco0921-20* operon et complemented by the *sco0921-20* operon or *sco0921* or *sco0920* alone whose expression was put under the control of the strong ermE* promoter. Strains were grown for 72 h at 28°C on solid R2YE medium with glucose (50 mM) as the main carbon source and containing 1 mM phosphate (Pi limitation). **(A)** LC/Corona-CAD analysis of the total lipid content of *S. lividans* TK24 (white histograms), *S. lividans* TK24 deleted for the *sco0921-20* operon (black histograms), *S. lividans* TK24 deleted for the *sco0921-20* operon complemented by the *sco0921-20* operon (dark gray histograms), *S. lividans* TK24 deleted for the *sco0921-20* operon complemented by *sco0921* (medium gray histograms) and *S. lividans* TK24 deleted for the *sco0921-20* operon complemented by *sco0920* (light gray histograms). **(B)** LC/Corona-CAD analysis of the total lipid content of *S. coelicolor* M145 (white histograms), *S. coelicolor* M145 deleted for the *sco0921-20* operon (black histograms), *S. coelicolor* M145 deleted for the *sco0921-20* operon complemented by the *sco0921-20* operon (dark gray histograms), *S. coelicolor* M145 deleted for the *sco0921-20* operon complemented by *sco0921* (medium gray histograms) and *S. coelicolor* M145 deleted for the *sco0921-20* operon complemented by *sco0920* (light gray histograms). TAG, triacylglycerol; DAG, diacylglycerol (1,2 or 1,3); FA, fatty acids; MAG, monoacylglycerol; PE, phosphatidylethanolamine; OL, ornithine lipids; PI, phosphatidylinositol; Ac-PIM2, acetylated phosphatidylinositol mannoside 2; CL, cardiolipid. Means values are shown as histograms with error bars representing standard error. Means sharing a letter are not significantly different (*P* > 0.05; Tukey-adjusted comparisons).

In *SL* grown in condition of Pi limitation, the deletion of the *sco0921-20* operon resulted in a 16.8 fold reduction of the OL content (Figure 3A). The reduction in OL content was correlated with a slightly higher TAG (1.1 fold) content accompanied by slightly lower content in biosynthetic intermediates such FA (1.2 fold) and MAG (1.2 fold) compared to the original strain (Figure 3A). This suggested that in absence of OL synthesis, acyl chains (FA) usually used for OL synthesis are being transferred by acyltransferases present in the bacteria, onto glycerol3P, MAG or DAG to synthesize TAG. The complementation of this mutant by the *sco0921-20* operon restored OL and TAG content to WT level but was also correlated with a significant increase in PE (1.8 fold) and CL (2.2 fold) content compared to the deletion mutant. This was accompanied by a lower content in the biosynthetic intermediates, FA (2.5 fold), MAG (3.2 fold), and 1,3-DAG (1.4 fold) (Figure 3A). Interestingly, the complementation of the deletion mutant by *sco0920* alone gave a lipid profile similar (except for PE) to that obtained with the complementation with the operon. In contrast, the complementation of the deletion mutant with *sco0921* alone of the deletion mutant had no impact on its PE, CL, TAG, 1,3-DAG, 1,2-DAG and MAG content (Figure 3A). Only a slight decrease in the FA content of this strain was noticed compared to the deletion mutant. These results unexpectedly suggested that SCO0920 might have both a *N*- and a broad *O*-acyltransferase activity and was able to transfer acyl chains to ornithine to synthetize lyso-ornithine (*N* acylation) as well as to achieve the *O*-acylation of lyso-ornithine and of other backbones such as precursors of cardiolipin.

**FIGURE 4:**
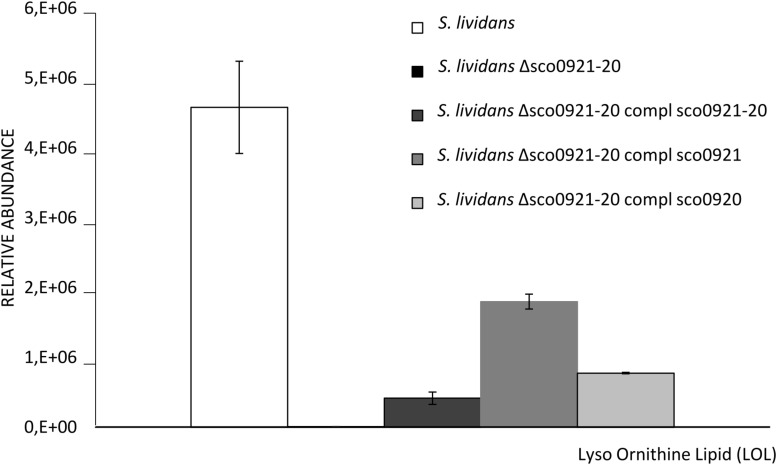
Mass spec analysis of the total lyso ornithine content of *S. lividans* TK24 *wt* (white histograms), *S. lividans* TK24 deleted for the *sco0921-20* operon (black histograms invisible) and this deletion mutant complemented by the *sco0921-20* operon (dark gray histograms), *sco0921* alone (medium gray histograms) or *sco0920* alone (light gray histograms). Strains were grown for 72 h at 28°C on solid R2YE medium with glucose (50 mM) as the main carbon source and containing 1 mM phosphate (Pi limitation). Means values are shown as histograms with error bars representing standard error.

In order to confirm this hypothesis lyso-ornithine was assayed in our strains by LC/MS (Figure 4). This study confirmed that LOL were detected in the *wt* strain but not in the *sco0921-20* deletion mutant whereas LOL were indeed detected in the mutant strain complemented by *sco0920* as well as by *sco0921.*This confirmed that SCO0920 bears *N*-acyltransferase activity besides its broad *O*-acyltransferase activity. The 2.3 lower LOL content of the mutant strain complemented by *sco0920* compared to that complemented by *sco0921* could be interpreted as the rapid conversion of LOL into OL by the *O*-acyltransferase function of *sco0920*. The presence of LOL in the mutant strain complemented by *sco0921* but the absence of OL in this strain confirmed that SCO0921 is a strict *N*-acyltransferase and has no *O*-acyltransferase function. The 11 fold lower LOL content of the mutant strain complemented by the *sco0921-20* operon compared to the *wt* strain might seem surprising (Figure 4). In the *wt* strain the operon is expressed from its own regulated promoter whereas in the complemented mutant strain the operon is present at an ectopic position and its expression is under the control of the strong *ermE*^∗^ promoter. We thus hypothesized that in the *wt* strain the level of expression of the first gene of the operon, *sco0921*, would be higher than that of the second one, *sco0920*. Consequently, in the *wt* strain the conversion of LOL into OL would be slowed down leading to LOL accumulation. In the complemented mutant both genes would be expressed at a high level from the strong *ermE*^∗^ promoter, facilitating the rapid conversion of LOL into OL.

In *SC*, the deletion of the *sco0921-20* operon was correlated with a reduced content (1.8 fold) of the weak OL content of the strain and with higher TAG (1.3 fold) and PE (1.7 fold) content (Figure 3B). This suggested that acyl chains not used for OL synthesis are being used for the biosynthesis of other lipids (TAG and PE) in this strain as in *SL*. This mutant was complemented by a unique ectopic copy of the *sco0921-20* operon, or of *sco0920* or of *sco0921* alone. The complementation of the deletion mutant by the *sco0921-20* operon was correlated with a 3.7 fold increase in the OL content. In contrast to what was seen for *SL*, the complementation of the *SC* deletion mutant with *sco0920* alone had no impact on the OL content. This might be due to the fact that the OL content of the deletion mutant of *SC* (Figure 3B) is very weak and its quantification is thus somehow unreliable being too close of the detection limit. The complementation of the *SC* deletion mutant with *sco0921* alone was not correlated either with an increase in the OL content but was correlated with a slight increase in 1,2-DAG (1.2 fold) and TAG (1.2 fold) content and a decrease in MAG content (1.2 fold) compared to the deletion mutant.

### Impact of the Over-Expression of the *sco0921-20* Operon, of *sco0921* or of *sco0920* on the Lipidome of *S. lividans* and *S. coelicolor*

An extra copy of the *sco0921-20* operon or of *sco0921* or of *sco0920* alone expressed under the control of the *ermE*^∗^ promoter (Siegl et al., 2013) was introduced at an ectopic position at the pSAM2 attB integration site (Boccard et al., 1989) in the genome of *S. lividans* and *S. coelicolor*. These two strains still have a native copy of this operon under the control of its own promoter but in *S. coelicolor* this native copy was shown to be weakly expressed (Figure 2A) so that is mainly the impact of the ectopic copies of these genes that is seen in this strain.

**FIGURE 5:**
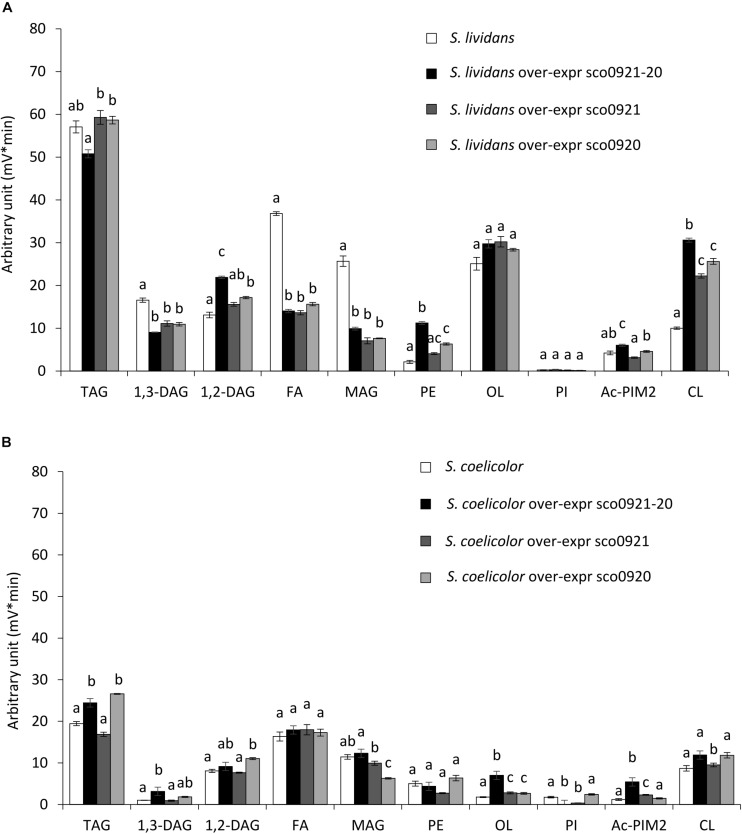
LC/Corona-CAD analysis of the total lipid content of *wt* strains *S. lividans* TK24 and *S. coelicolor* M145 and of these strains over-expressing under the control of the strong ermE* promoter, the *sco0921-20* operon or *sco0921* or *sco0920* alone. The strains were grown for 72 h at 28°C on solid R2YE medium with glucose (50 mM) as the main carbon source and containing 1mM phosphate (Pi limitation). **(A)** LC/Corona-CAD analysis of total lipid content of *S. lividans* TK24 (white histograms), *S. lividans* TK24 over-expressing the *sco0921-20* operon (black histograms), *S. lividans* TK24 over-expressing *sco0921* (dark gray histograms) and *S. lividans* TK24 over-expressing *sco0920* (light gray histograms). **(B)** LC/Corona-CAD analysis of the total lipid content of *S. coelicolor* M145 (white histograms), *S. coelicolor* M145 over-expressing the *sco0921-20* operon (black histograms), *S. coelicolor* M145 containing an extra copy of *sco0921* (dark gray histograms) and *S. coelicolor* M145 containing an extra copy of *sco0920* (light gray histograms). TAG, triacylglycerol; DAG, diacylglycerol (1,2 or 1,3); FA, fatty acids; MAG, monoacylglycerol; PE, phosphatidylethanolamine; OL, ornithine lipids; PI, phosphatidylinositol; Ac-PIM2, acetylated phosphatidylinositol mannoside 2; CL, cardiolipid. Means values are shown as histograms with error bars representing standard error. Means sharing a letter are not significantly different (*P* > 0.05; Tukey-adjusted comparisons).

**FIGURE 6:**
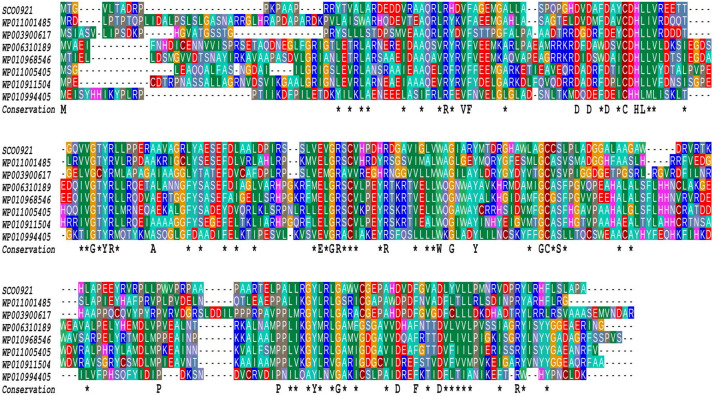
Multiple sequence alignment of SCO0921 with representative members of COG3176, a Conserved Domain Database (CDD) family of putative hemolysins (Lu et al., 2020). The proteins aligned originate from Ralstonia (WP011001485), Mycobacterium tuberculosis (WP003900617), Agrobacterium (WP006310189, Rhizobiales (WP010968546), Brucella (WP011005405), Mesorhizobium (WP010911504), and Nostocaceae (WP010994405). The amino acids identical in all aligned sequences are indicated by a one letter code under the alignment whereas asterisks indicate conserved similarities.

In *SC*, the over-expression of the *sco0921-20* operon led to an almost total disappearance of PI that was accompanied by a slight increase in the TAG (1.2 fold), 1,3-DAG (2.9 fold), OL (3.6 fold), and Ac-PIM2 content (3.7 fold) compared to the original strain (Figure 5B). The over-expression of *sco0921* alone was also correlated with a strong reduction (4.1 fold) in the PI content that, in contrast with the over-expression of the operon, was not correlated with an increase in the TAG nor 1,3-DAG content but was correlated with a slightly higher content in OL (1.4 fold) and Ac-PIM2 (1.6 fold) content. This suggested that SCO0921 might be responsible for the disappearance of PI. Since SCO0921 bears similarities with hemolysins (Figure 6) and hemolysins often bears phospholipase C activity (Maheswaran and Lindorfer, 1967; Pal et al., 1997), we propose that SCO0921 hydrolyzes PI into 1,2-DAG and inositol P. This specific lipid would thus somehow constitute a phosphorus reserve. The over-expression of *sco0920* alone was correlated with a clear increase in TAG (1.3 fold) and 1,2-DAG (1.3 fold) content and a decrease in MAG content (1.7 fold). This confirmed the O-acyltransferase function of this enzyme that otherwise had no impact on the PI content.

In *SL*, the presence of an extra ectopic copy of the *sco0921-20* operon led to an increase in CL (2.9 fold), PE (4.4 fold), Ac-PIM2 (1.2 fold), and 1,2-DAG (1.6 fold) content that was correlated with a decrease in FA (2.5 fold), MAG (2.4 fold), and 1,3-DAG (1.8 fold) content compared to the original strain (Figure 5A). The presence of an extra ectopic copy of *sco0920* alone led to an increase in CL (2.4 fold), PE (2.4 fold), and 1,2-DAG (1.2 fold) content that was correlated with a decrease in FA (2.3 fold), MAG (3.2 fold), and 1,3-DAG (1.4 fold) content (Figure 5A). The presence of an extra ectopic copy of *sco0921* alone was correlated with an increase in CL (2.1 fold) content and a decrease in FA (2.6 fold), MAG (3.1 fold), and 1,3-DAG (1.4 fold) content (Figure 5A). One notices the three *S. lividans* strains over-expressing the *sco0921-20* operon, *sco0921* or *sco0920* alone showed similar trends. The native operon being highly expressed in this strain, the absence of one or the other function in the strains containing an extra ectopic copy of *sco0920* or *sco0921* is automatically compensated by the copy present in the native operon.

### Impact of the Over-Expression of the *sco0921-20* Operon or of *sco0921* or *sco0920* Alone on Actinorhoddin Production in *S. coelicolor*

**FIGURE 7:**
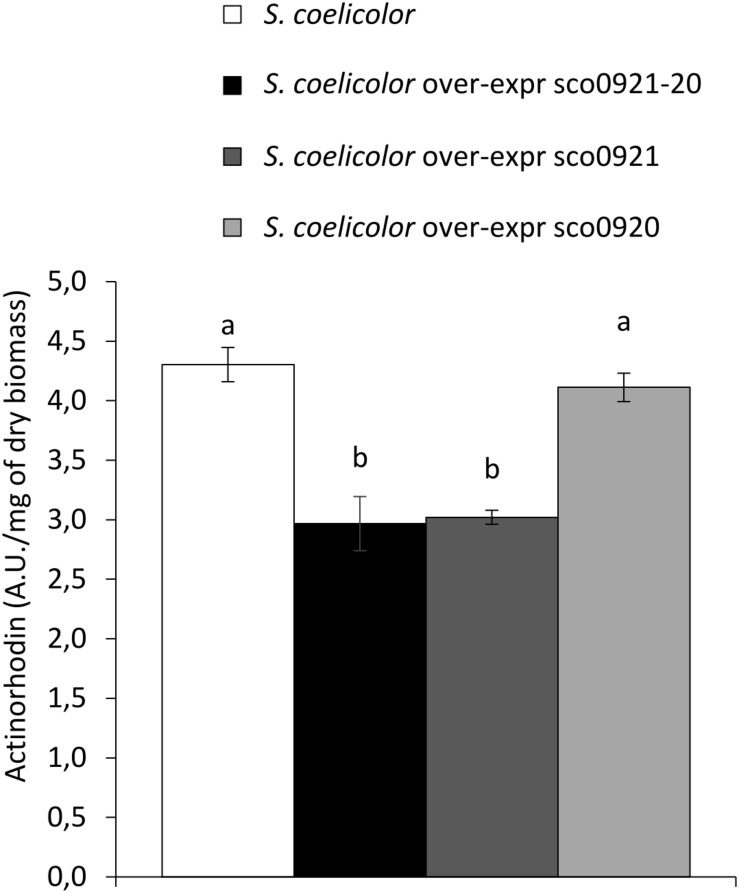
Quantification of the extracellular actinorhodin in the culture medium of *S. coelicolor* (white histograms), *S. coelicolor* over-expressing the *sco0921-20* operon (black histograms), or *sco0921* (dark gray histograms) or *sco0920* (light gray histograms) alone. The strains were grown for 72 h at 28°C on solid agar modified R2YE medium with no phosphate added (conditions of phosphate limitation). Means values are shown as histograms with error bars representing standard error. Means sharing a letter are not significantly different (*P* > 0.05; Tukey-adjusted comparisons).

In Sandoval-Calderón et al. (2015), the authors mentioned that the disruption of *sco0921* by a transposon led to an early and enhanced ACT biosynthesis. The complementation of this mutant by the whole operon restored ACT production to wild type level but the authors did not provide any explanation for this interesting observation. We think that this observation is fully consistent with our hypothesis that SCO0921 acts as a phospholipase C involved in the cleavage of PI into inositol P and DAG. Inositol P would be dephosphorylated by broad specificity phosphatases induced in condition of phosphate limitation to provide phosphate. This Pi supply might be responsible for the observed repression of ACT biosynthesis. To verify this hypothesis, the *sco0921-20* operon, *sco0921* or *sco0920* alone, expressed from the strong *ermE*^∗^ operon, were introduced into the native strain of *SC* and extracellular ACT production of these strains was assayed. Results shown in Figure 7 indicated that the over-expression of the *sco0921-20* operon or of *sco0921* alone was correlated with a reduction of ACT biosynthesis compared to the wild strain whereas the over-expression of *sco0920* alone had no impact on ACT production. We proposed that the negative impact that *sco0921* over-expression has on ACT production might be due to its phospholipase C-like activity that indirectly allows the liberation of the Pi trapped into PI.

## Discussion

In this issue we demonstrated that in condition of phosphate (Pi) proficiency the phospholipid (PE, CL, and PI) content of *S. coelicolor* (*SC*) was lower than that of *S. lividans* (*SL*) and varied little with Pi availability. In contrast, the phospholipid content of *SL* varies greatly with Pi availability being was much lower in Pi limitation than in Pi proficiency. The limited variation of the phospholipid (PL) content in *SC* according to Pi availability might be due its characteristic oxidative metabolism that is known to consume acetylCoA and thus reduces acetylCoA availability (Esnault et al., 2017). It might also be due to the weak expression of the high (*pstSCAB*) and low (*sco4138/pitH2*) affinity phosphate transporters in this strain (Santos-Beneit et al., 2008) that is under the positive control of PhoR/PhoP that is poorly expressed in *SC* (Millan-Oropeza et al., 2020). The poor expression of these transporters in *SC* likely leads to a severe phosphate limitation.

In contrast the content in the phosphate-free OL of both strains increased in condition of Pi limitation. We demonstrated that the biosynthesis of OL requires mainly SCO0920, an enzyme encoded by the second gene of the *sco0921-20* operon. SCO0920 was shown to bear both *N*- and *O*-acyltransferase activities being thus more similar to OlsF of *Serratia proteamaculans*, *Vibrio cholerae*, and *Klebsiella pneumoniae* that also bears both *N*- and *O*-acyltransferase function (Vences-Guzmán et al., 2015; Barbosa et al., 2018; MetaCyc olsF, 2020) than to OlsA, a specific *O*-acyltransferase (Weissenmayer et al., 2002; Gao et al., 2004; Sandoval-Calderón et al., 2015; Sohlenkamp, 2016). Furthermore, our data indicated that SCO0920 is an *O*-acyltransferase with a broad substrate specificity since it is able to transfer acyl chains onto various backbones, besides LOL, such as precursors of TAG, PE, or CL.

Our experiments confirmed the previously reported strict *N*-acyltransferase activity of SCO0921 (Sandoval-Calderón et al., 2015) since the complementation the *sco0921-20* deletion mutant by *sco0921* alone yielded LOL but not OL. Interestingly, the over-expression of *sco0921* in *SC* was correlated with an almost total disappearance of PI (Figure 5B). Since SCO0921 bears similarities with hemolysins that usually bear phospholipase C activity (Maheswaran and Lindorfer, 1967; Pal et al., 1997), we proposed that SCO0921 has phospholipase C activity and hydrolyzes PI into 1,2-DAG and inositol phosphate. The higher content in 1,2-DAG, in its isomerized form 1,3-DAG and in TAG directly derived from these precursors, in *SC* deleted for the operon and complemented by SCO0921 alone, is consistent with this hypothesis (Figure 3B). Similarly, the 53.5 fold lower PI content of *SL* compared to *SC* (Figure 1c) in conditions of Pi limitation that likely results from the cleavage of this specific lipid by SCO0921 whose expression is 13 fold higher in *SL* than in *SC* in condition of Pi limitation (Figure 2A) is also consistent with this hypothesis. Interestingly, a phospholipase C was also shown to be necessary for lipid remodeling during phosphorus limitation in the Gram negative bacteria, *Sinorhizobium meliloti* (Zavaleta-Pastor et al., 2010).

Inositol phosphate generated by the degradation of PI by SCO0921 is predicted to be dephosphorylated by various phosphatases induced in condition of phosphate scarcity, to provide phosphate. This specific lipid would thus constitute somehow a phosphorus reserve. The phosphate resulting indirectly from its degradation would repress actinorhoddin (ACT) production (Martín, 2004). Indeed ACT production was shown to be lower in the *SC* strains over-expressing the *sco0921-20* operon or *sco0921* alone in than in the *wt* strain or in the strain over-expressing *sco0920* alone (Figure 7). Consistently, Sandoval-Calderón et al. (2015) reported that ACT production occurred earlier and was enhanced in the *SC* strain deleted for the *sco0921-20* operon.

In conclusion, we wish to stress that, to our knowledge, our work is the first one to demonstrate that the combination of genetic and lipidomic approaches (Abreu et al., 2017) is an extremely useful and powerful approach to elucidate the *in vivo* function of enzymes involved in lipid metabolism.

## Data Availability Statement

The original contributions presented in the study are included in the article/Supplementary Material, further inquiries can be directed to the corresponding author/s.

## Author Contributions

CL, SA, and TD contributed to experimental materials and reagents, conception, execution of the experiments, data management, and reporting. MD executed the experiments. SW carried out protein alignements. M-JV, PC, SA, and CL were involved in the interpretation of the data and literature review. M-JV wrote the manuscript and provided financial support for the work. PC provided the tools and instruments that were vital for the project. PC, SA, SW and CL reviewed the manuscript before submission for both grammar and intellectual content. All authors contributed to the article and approved the submitted version.

## Conflict of Interest

The authors declare that the research was conducted in the absence of any commercial or financial relationships that could be construed as a potential conflict of interest.
