# Supplementary material for: Impact of Phosphate Availability on Membrane Lipid Content of the Model Strains, Streptomyces lividans and Streptomyces coelicolor
Source: Front Microbiol. 2021 Feb 22;12:623919. doi: 10.3389/fmicb.2021.623919 (PMC7937720; doi:10.3389/fmicb.2021.623919)
Supplement: Supplementary file 2 [file Table_2.docx]

**Table S2: Strains and plasmids used in this study**

| **Strain or plasmid** | **Relevant property(ies)**^¶^ | | **Reference** |
| --- | --- | --- | --- |
| *S. coelicolor* strains | | | |
| M145 WT | | Wild type strain. | [1] |
| M145 ∆*sco0921-20*::Ω *apra^R^* | | WT M145 containing apramycin resistance cassette (*aac(3)-IV*) in place of *sco0921-20* operon. | This study |
| M145 *att*pSAM2::pOSV557-*sco0921-20* | | WT M145 containing the plasmid pOSV557-*sco0921-20* integrated at pSAM2 *att*B site [2] | This study |
| M145 *att*pSAM2::pOSV557-*sco0921* | | WT M145 containing the plasmid pOSV557-*sco0921* integrated at pSAM2 *att*B site [2]. | This study |
| M145 *att*pSAM2::pOSV557-*sco0920* | | WT M145 containing the plasmid pOSV557-*sco0920* integrated at pSAM2 *att*B site [2]. | This study |
| M145 ∆*sco0921-20*::Ω *apra^R^*  *att*pSAM2::pOSV557-*sco0921-20* | | WT M145 containing apramycin resistance cassette (*aac(3)-IV*) in place of *sco0921-20* operon and the plasmid pOSV557-*sco0921-20* integrated at pSAM2 *att*B site [2]. | This study |
| M145∆ *sco0921-20*::Ω *apra^R^*  *att*pSAM2::pOSV557-*sco0921* | | WT M145 containing apramycin resistance cassette (*aac(3)-IV*) in place of *sco0921-20* operon and the plasmid pOSV557-*sco0921* integrated at pSAM2 *att*B site [2]. | This study |
| M145 ∆*sco0921-20*::Ω *apra^R^*  *att*pSAM2::pOSV557-*sco0920* | | WT M145 containing apramycin resistance cassette (*aac(3)-IV*) in place of *sco0921-20* operon and the plasmid pOSV557-*sco0920* integrated at pSAM2 *att*B site [2]. | This study |
| *S. lividans* strains | | | |
| TK24 WT | | Wild type strain. | [3] |
| TK24 ∆*sco0921-20*::Ω *apra^R^* | | WT TK24 containing apramycin resistance cassette (*aac(3)-IV*) in place of *sco0921-20* operon. | This study |
| TK24 *att*pSAM2::pOSV557-*sco0921-20* | | WT TK24 containing the plasmid pOSV557-*sco0921-20* integrated at pSAM2 *att*B site [2]. | This study |
| TK24 *att*pSAM2::pOSV557-*sco0921* | | WT TK24 containing the plasmid pOSV557-*sco0921* integrated at pSAM2 *att*B site [2]. | This study |
| TK24 *att*pSAM2::pOSV557-*sco0920* | | WT TK24 containing the plasmid pOSV557-*sco0920* integrated at pSAM2 *att*B site [2]. | This study |
| TK24 ∆*sco0921-20*::Ω *apra^R^*  *att*pSAM2::pOSV557-*sco0921-20* | | WT TK24 containing apramycin resistance cassette (*aac(3)-IV*) in place of *sco0921-20* operon and the plasmid pOSV557-*sco0921-20* integrated at pSAM2 *att*B site [2]. | This study |
| TK24 ∆*sco0921-20*::Ω *apra^R^*  *att*pSAM2::pOSV557-*sco0921* | | WT TK24 containing apramycin resistance cassette (*aac(3)-IV*) in place of *sco0921-20* operon and the plasmid pOSV557-*sco0921* integrated at pSAM2 *att*B site [2]. | This study |
| TK24 ∆*sco0921-20*::Ω *apra^R^*  *att*pSAM2::pOSV557-*sco0920* | | WT TK24 containing apramycin resistance cassette (*aac(3)-IV*) in place of *sco0921-20* operon and the plasmid pOSV557-*sco0920* integrated at pSAM2 *att*B site [2]. | This study |
| *E. coli* strains | | | |
| DH5α | | General cloning strain. | [4] |
| ET12567 pUZ8002 | | Strain used for conjugation between *E. coli* and *Streptomyces.* | [5] |
| Plasmids | | | |
| pOSV400 | | ColE1*, oriT, lacZ’*, *hph*  This plasmid is replicative in *E. coli* but not in *S. coelicolor* and *S.* *lividans*. | [6] |
| pOSV400-UD-*sco0921-20-apra^R^* | | pOSV400 containing upstream (1.3 kb) and downstream regions of *sco0921-20* operon (1.2 kb) with apramycin resistance cassette (*aac(3)-IV*) between them. | This study |
| pOSV557 | | ColE1*, oriT, attP*, *int, hph, amp^R^*  This plasmid is identical to pOSV010 [7] including a constitutive promoter *ermE** [8]. It is replicative in *E. coli* but integrative in *S. coelicolor* and *S. lividans*. | Unpublished data |
| pOSV557-*sco0921-20* | | pOSV557 harboring *sco0921-20* gene under the control of the constitutive promoter *ermE** [8]. | This study |
| pOSV557-*sco0921* | | pOSV557 harboring *sco0921* gene under the control of the constitutive promoter *ermE** [8]. | This study |
| pOSV557-*sco0920* | | pOSV557 harboring *sco0920* gene under the control of the constitutive promoter *ermE** [8]. | This study |

^¶^ WT, Wild Type; apra^R^, resistance to apramycin, amp^R^, resistance to ampicillin, ColE1, origin of replication in *E. coli* (not functional in *S. coelicolor* and *lividans*); *oriT*, origin of transfer; *lacZ’*, gene encoding the LacZα; *hph* : gene encoding the resistance to hygromycin; *attP*, pSAM2 attachment site; *int*, pSAM2 conjugative integrase gene.

**References**

1. Bentley, S.D. Complete genome sequence of the model actinomycete Streptomyces coelicolor A3(2. *Nature* **2002**, *417*, 141–147.

2. Boccard, F.; Smokvina, T.; Pernodet, J.L.; Friedmann, A.; Guérineau, M. The integrated conjugative plasmid pSAM2 of Streptomyces ambofaciens is related to temperate bacteriophages. *EMBO J.* **1989**, *8*, 973–980.

3. Ruckert, C. Complete genome sequence of Streptomyces lividans TK24. *J Biotechnol* **2015**, *199*, 21–22.

4. Hanahan, D. Studies on transformation of Escherichia coli with plasmids. *J. Mol. Biol.* **1983**, *166*, 557–580, doi:10.1016/s0022-2836(83)80284-8.

5. Gust, B.; Chandra, G.; Jakimowicz, D.; Yuqing, T.; Bruton, C.J.; Chater, K.F. Lambda red-mediated genetic manipulation of antibiotic-producing Streptomyces. *Adv. Appl. Microbiol.* **2004**, *54*, 107–128, doi:10.1016/S0065-2164(04)54004-2.

6. Boubakri, H.; Seghezzi, N.; Duchateau, M.; Gominet, M.; Kofroňová, O.; Benada, O.; Mazodier, P.; Pernodet, J.-L. The Absence of Pupylation (Prokaryotic Ubiquitin-Like Protein Modification) Affects Morphological and Physiological Differentiation in Streptomyces coelicolor. *J. Bacteriol.* **2015**, *197*, 3388–3399, doi:10.1128/JB.00591-15.

7. Juguet, M.; Lautru, S.; Francou, F.-X.; Nezbedová, S.; Leblond, P.; Gondry, M.; Pernodet, J.-L. An iterative nonribosomal peptide synthetase assembles the pyrrole-amide antibiotic congocidine in Streptomyces ambofaciens. *Chem. Biol.* **2009**, *16*, 421–431, doi:10.1016/j.chembiol.2009.03.010.

8. Bibb, M.J.; Janssen, G.R.; Ward, J.M. Cloning and analysis of the promoter region of the erythromycin resistance gene (ermE) of Streptomyces erythraeus. *Gene* **1985**, *38*, 215–226, doi:10.1016/0378-1119(85)90220-3.
